# Multi-channel router and logic NAND gate from multiple Autler–Townes splitting controlled by phase transition

**DOI:** 10.1039/d0ra01379j

**Published:** 2020-04-17

**Authors:** Faizan Raza, Irfan Ahmed, Habib Ullah, Hammad-ul Wahab, Ubaid Khan, Yanpeng Zhang

**Affiliations:** Key Laboratory for Physical Electronics and Devices of the Ministry of Education, Shaanxi Key Lab of Information Photonic Techniques, Xi'an Jiaotong University Xi'an 710049 China ypzhang@mail.xjtu.edu.cn; Department of Physics, City University of Hong Kong Hong Kong iahmed8-c@my.cityu.edu.hk; Electrical Engineering Department, Sukkur IBA Sukkur 65200 Sindh Pakistan

## Abstract

For the first time, we investigated the electric-dipole transition dependent primary and secondary temporal Autler–Townes (TAT)-splitting of a hybrid signal (fluorescence and Stokes) in Pr^3+^:YPO_4_. We compared the TAT-splitting in different phases (pure tetragonal (T), pure hexahedral (H), (T + H)-phase, and (H + T)-phase) of the Pr^3+^:YPO_4_ crystal. The TAT-splitting in the (H + T)-phase was observed to be stronger than that in other phases, while the Pr^3+^ ion had stronger dressing than the Eu^3+^ ion in the host material of YPO. Furthermore, we observed that the ratio of primary and secondary TAT-splitting can be controlled by the single and double dressing effect using the power and detuning of employed laser fields. In our experiment, we observed that secondary splitting from secondary dressed levels can only be observed at the resonance wavelength in the three-level system. Based on the results, we proposed a model for a multi-channel optical router and logic NAND gate. The routing action results from primary and secondary TAT-splitting, while the NAND gate was realized by the primary dressed states.

## Abstract 2

For the first time, we investigated the electric-dipole transition dependent primary and secondary temporal Autler–Townes (TAT)-splitting of a hybrid signal (fluorescence and Stokes) in Pr^3+^:YPO_4_.

## Graphical abstract

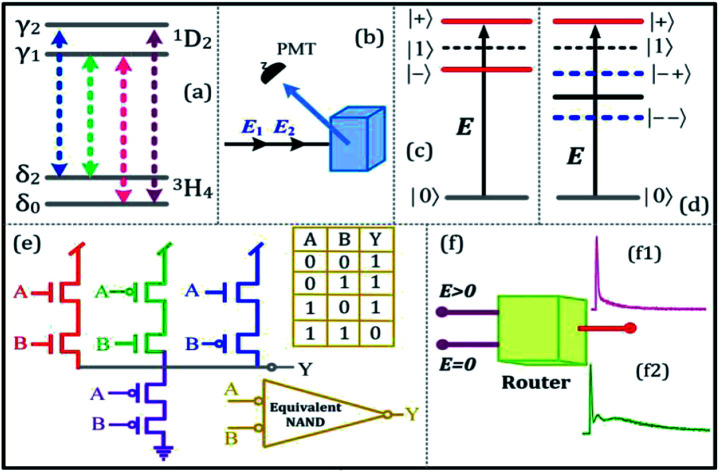

## Introduction

Physicists have achieved incredible progress in understanding and controlling the quantum coherence excitation and coherence transfer in atomic gases. These processes lead to numerous famous and intriguing physical phenomena, such as electromagnetically induced transparency (EIT)^1^ and spontaneous-parametric four-wave mixing (SP-FWM) under EIT conditions.^2^ The SP-FWM configuration with rubidium (Rb) atomic vapors is considered as an ideal quantum system due to the high imaging contrast, long coherence time (∼ns), and narrow spectral linewidth (∼MHz).^3^ However, it is less likely to integrate such systems with atomic vapors for the development and production of quantum technologies. In Pr^3+^-doped yttrium orthophosphate (YPO_4_), *i.e.*, Pr^3+^:YPO_4_, the “atom-like” properties of the dopant in which the atomic coherence can be induced when interacting with multiple laser beams is similar to other rare-earth-doped crystals.^4–6^ Unlike atomic gases, Pr^3+^:YPO_4_ can be used in integrated quantum circuits due to their solid crystal structure and induced coherence. Coherence excitation has been explored mostly in doped crystals such as Eu^3+^:YPO_4_, Pr^3+^:YPO_4_, and Pr^3+^:Y_2_SiO_5_, for improving lifetime, coherence time, spectral bandwidth, and the induction of non-classic behaviour. The Autler–Townes (AT) splitting of multi-order fluorescence (FL) has been investigated in numerous atomic-like media.^7,8^ In atomic-like media, the lifetime of FL processes can be controlled by the dressing effect, which can be adjusted by the power or detuning of laser fields.^9^ Recent progress has widely explored solid-state atomic coherent materials with EIT,^10^ reduction of optical velocity,^11^ optical storage based on all-optical routing,^12^ coherent storage of light pulses,^13^ and optical read and write information.^14^

Eu^3+^ and Pr^3+^ ions are more sensitive to the site symmetry and the surrounding crystal-field of the host material than other crystal ions,^15,16^ which makes them an attractive material for important applications such as in scintillation detectors, medical imaging, display devices^17,18^ while demonstrating a high chemical and thermal stability.^19,20^ In this regard, a double-cascade dressed optical metal oxide semiconductor field-effect transistor is realized by exploiting the enhancement and suppression in different phases of Eu^3+^:YPO_4_ and Pr^3+^:YPO_4_ crystals.^21^ YPO_4_ crystallizes with the zircon structure (xenotime-type) with a tetragonal symmetry (*a* = *b* = 0.6894 nm and *c* = 0.6027 nm) and space group *I*4_1_/*amd*,^22^ where the site symmetry for Y^3+^ ions is the *D*_2d_ point-group.^23^ The structure can be described as chains parallel to the *c*-axis of the corner-sharing structural units built of (YO_8_) dodecahedron and a (PO_4_) tetrahedron linked together by an edge.^24^ The YPO_4_ matrix has excellent optical and physical properties such as a large indirect bandgap (∼8.6 eV), high dielectric constant (∼7 eV), refractive index (∼1.72), high melting point (∼160 °C), and phonon energy (∼1080 cm^−1^).^25^

In this study, we investigated the relationship between temporal AT-splitting (TAT-splitting) and excitation spectra of the mixed-phase (much hexagonal (H) + less T) Pr^3+^:YPO_4_ crystal. We observed that the secondary TAT-splitting is very sensitive to the electric dipole transitions, which can be controlled through the wavelength and power of laser beams. Based on our results, we proposed a model of a multi-channel optical router and logic NAND gate controlled by a laser power.

## Experiment setup

**Fig. 1:**
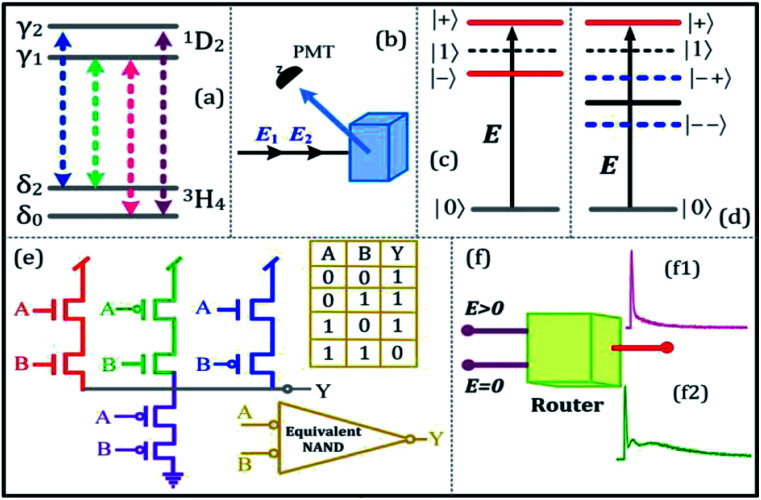
(a) Energy level of Pr^3+^:YPO_4_. (b) Experimental setup. (c) Single dressed energy level. (d) Double dressed energy level. (e) The schematic diagram of the MOSFET logic “NAND” gate, where (*A*, *B*) and *Y* is the input and output of proposed equivalent logic NAND gate, respectively. (f) The schematic diagram of the multi-channel router.

In this experiment, the sample of Pr^3+^:YPO_4_ crystal was held in a cryostat (CFM-102) maintained at 77 K by flowing liquid nitrogen. Fig. 1(a) shows a fine structure energy level of Pr^3+^:YPO_4_. Fig. 1(b) shows the schematic of the experimental setup where the photomultiplier tube (PMT) is placed to detect the generated Stokes (*E*_S_) and fluorescence (FL) hybrid signal under phase-matched four-wave mixing. Two dye lasers (narrow scan with a line width of 0.04 cm^−1^) were pumped by an injection-locked single-mode Nd:YAG laser (Continuum Powerlite DLS 9010, 10 Hz repetition rate, 5 ns pulse width), which were used to generate the pumping fields *E*_1_ (*ω*_1_, *Δ*_1_) and *E*_2_ (*ω*_2_, *Δ*_2_) with frequency detuning of *Δ*_*i*_ = *Ω*_*mn*_ − *ω*_*i*_, where *Ω*_*mn*_ is the corresponding atomic transition frequency between levels |*m*〉 and |*n*〉. *ω*_*i*_ (*i* = 1, 2) is the laser frequency. Fig. 1(c) and (d) show single and double dressed energy levels, respectively.

By opening the field *E*_1_, *E*_S_ was generated in a two-level system with phase matching condition 
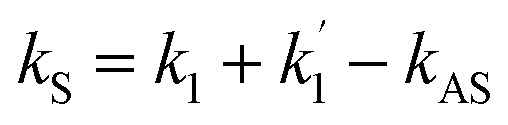
. The density matrix elements of *E*_S_ from a mixed (H + T)-phase Pr^3+^:YPO_4_*via* perturbation chain can be written as1
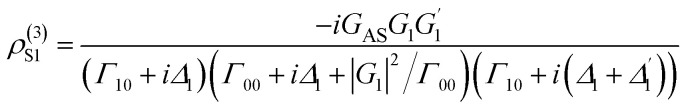
where *G*_*i*_ = *μ*_*i*_*E*_*i*_/*ℏ* is the Rabi frequency of field *E*_*i*_, with the electric dipole matrix elements *μ*_*ij*_ of levels |*i*〉 and |*j*〉 and *Γ*_*ij*_ = (*Γ*_*i*_ + *Γ*_*j*_)/2 is the transverse decay rate, where, *Γ*_*i*/*j*_ = *Γ*_pop_ + *Γ*_ion–spin_ + *Γ*_ion–ion_ + *Γ*_phonon_ − *Γ*_dressing_ + *Γ*_non-rad_. The density matrix of the accompanying FL can be written as

2 *ρ*^(2)^_11_ = −|*G*_1_|^2^/[(*d*_1_ + |*G*_1_|^2^/*Γ*_00_)(*Γ*_11_ + |*G*_1_|^2^/*d*_1_)]

where *d*_1_ = *Γ*_10_ + *iΔ*_1_. The lifetime of FL is given as *Γ*_FL_ = *Γ*_10_ + *Γ*_11_. The temporal intensity of FL is given as *I*(*t*) = *ρ*^(2)^_11_ exp(−*Γ*_FL_*t*). By opening *E*_1_ and *E*_2_ in a ∧-type three-level system (Fig. 1(c)), the third-order nonlinear density matrix elements of *E*_S_*via* the perturbation chain 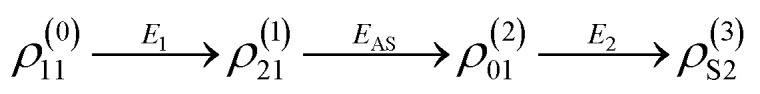 is given as3
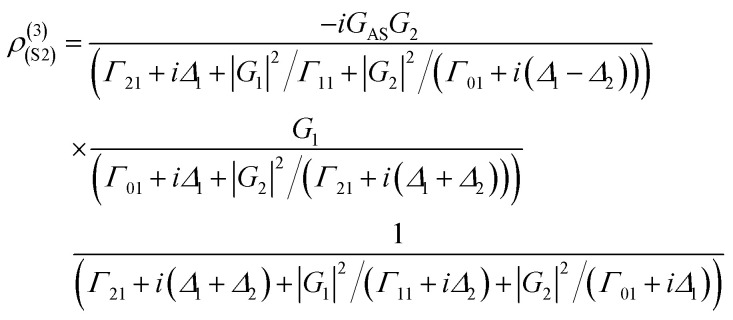


The lifetime of the Stokes signal can be written as *Γ*_S2_ = *Γ*_01_ + 2*Γ*_21_. Similarly, fourth-order FL in a ∧-type system *via* the pathway 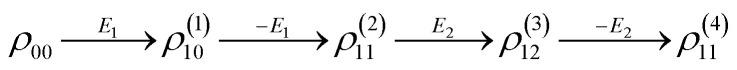 is4
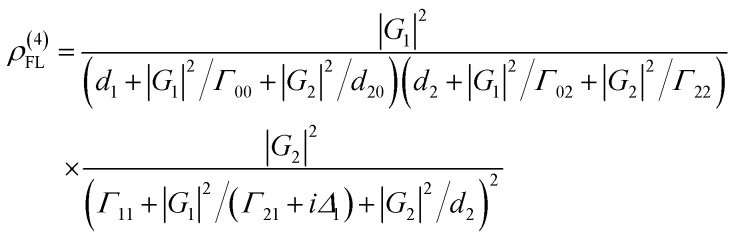


## Results and discussion

**Fig. 2:**
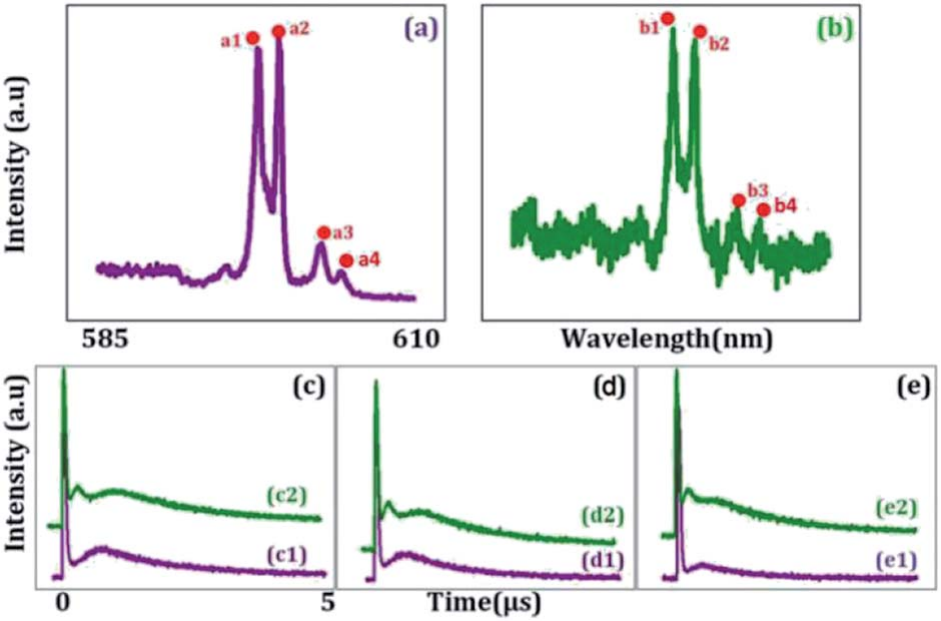
Shows spectral intensity signal obtained from Pr^3+^:YPO_4_ in (a) two-level atomic system when *E*_1_ is scanned from 585 nm to 620 nm (*E*_2_ is blocked), (b) ∧-type three-level system when *E*_1_ is scanned and *E*_2_ is set at resonance wavelength. (c1), (d1) and (e1) Measured time domain intensity of hybrid signal with *E*_1_ being at 595 nm, 596.7 nm and 600.0 nm, respectively, in a two level system. (c2), (d2) and (e2) Measured time domain intensity of hybrid signal with *E*_2_ being at 595 nm, 596.7 nm and 600.0 nm, respectively, while *E*_1_ is fixed at resonant wavelength.

Fig. 2(a) and (b) shows the excitation spectrum of the hybrid signal (FL + *E*_S_) measured from the (H + T)-phase Pr^3+^:YPO_4_ crystal in two-level and three-level ∧-type systems, respectively. The excitation spectrum in Fig. 2 is measured by scanning *E*_1_ from 585 nm to 610 nm by fixing the gate position (gate width = 100 ns) at a certain point (5 μs on the time-domain curve (Fig. 2(c)). In Fig. 2, four electric dipoles allowed transitions between the Stark levels of ^1^D_2_ and ^3^H_4_ are detected, whose energy levels are shown in Fig. 1(a). The four peaks a1/b1 (595.1 nm), a2/b2 (596.7 nm), a3/b3 (600.0 nm), and a4/b4 (601.5 nm) correspond to the electric-dipole allowed transitions between ^1^D_2_ (γ_2_) → ^3^H_4_ (δ_0_), ^1^D_2_ (γ_1_) → ^3^H_4_ (δ_0_), ^1^D_2_ (γ_2_) → ^3^H_4_ (δ_2_), and ^1^D_2_ (γ_1_) → ^3^H_4_ (δ_2_), respectively. Under the action of the crystal field of YPO and site symmetry (*D*_2_ + *D*_2d_) of the (H + T)-phase, the terms ^3^H_4_ (ground state) and ^1^D_2_ (excited state) under dipole-allowed transition can split into seven and four fine structure levels, as shown in Fig. 1(a).^26^ In our experiment, we observed only four peaks in (much hand less T)-phase YPO_4_ (Fig. 2(a)), which suggests that the site symmetry of (*D*_2_ + *D*_2d_) is not strong enough to completely lift the 2*J* + 1 degeneracy of the levels. It is worth mentioning here that each electric-dipole transition corresponds to different lifetimes and dressing effects. Fig. 2(c) shows the time-domain intensity of the hybrid signal detected at PMT in the two-level (Fig. 2(c1)) and ∧-type three-level (Fig. 2(c2)) systems. Fig. 2(c1) demonstrates the two peaks in the temporal intensity of the hybrid signal. The right peak is primary TAT-splitting attributed to the adiabatic population transfer between the dressed states, whereas the left peak is the contribution of (FL + *E*_S_) without the adiabatic population transfer. If we set |1〉 as the frequency reference point, the Hamiltonian for primary TAT-splitting can be written using 
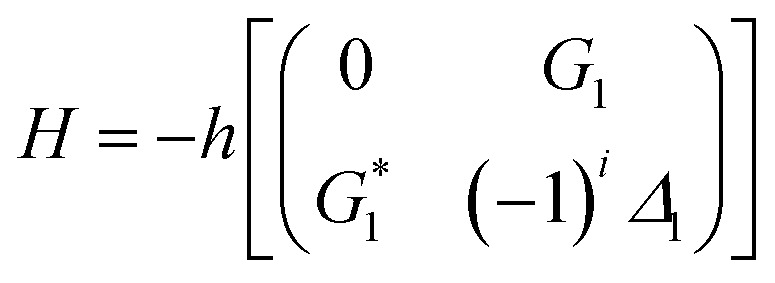
.^27^ From the equation *H*|±〉 = *λ*_±_|±〉, we can obtain *λ*_±_ = [*Δ*_1_ ± (*Δ*_12_ + 4|*G*_1_|^2^)^1/2^]/2. The splitting distance between |+〉 and |−〉 is *Δ*_±_ = *λ*_+_ − *λ*_−_ = (*Δ*_12_ + 4|*G*_1_|^2^)^1/2^. The primary splitting distance *Δ*_±_ is directly proportional to |*G*_1_|^2^. In the primary TAT-splitting, the left and right peaks of the time-intensity signal correspond to the dressed states |+〉 and |−〉, respectively, as shown in Fig. 1(d). When both *E*_1_ and *E*_2_ are turned on, the primary dressed level |−〉 is further split into two secondary dressed levels |−+〉 and |−−〉, as shown in Fig. 1(d). We named this as secondary splitting, whose Hamiltonian for the secondary TAT-splitting is 
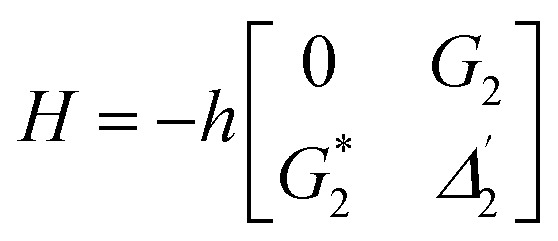
 (where 
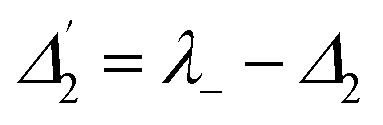
) and from the equation *H*′|−±〉 = *λ*_−±_|−±〉, we can obtain *Δ*_−±_ = *λ*_−+_ − *λ*_−−_ = [*Δ*_2_ ± (*Δ*_22_ + 4|*G*_2_|^2^)^1/2^]/2. At *Δ*_2_ ≡ 0, we can know that the increase in the |*G*_2_|^2^ can lead to an increase in the |*λ*_−_|. Using the above equations, we can derive the formula for TAT-splitting^8^*t*_AT_ ∝ 1/*Γ*_non-rad_ + *A* exp(*Δ*_±_/*K*_B_T). The secondary splitting distance of the right peak in Fig. 2(c2) is physically due to residual particles in |−+〉 transferring to |−−〉 through the phonon-assisted non-radiative transition. Acoustic phonons mainly determine this phonon-assisted non-radiative transition at low temperatures. The three peaks in the FL time-domain signal (Fig. 2(c2) and (d2)), from left to right, can be corresponded to the primary dressed state |+〉 and the secondary dressed states |−+〉 and |−−〉, respectively, as shown in Fig. 1(d). One can witness that in a ∧-type system, the time-domain signal has both secondary and primary TAT-splitting (Fig. 2(c2)), whereas only primary TAT-splitting was observed in the two-level system (Fig. 2(c1)). However, it is worth mentioning that primary TAT-splitting appears to be much stronger in the two-level system (Fig. 2(c1)). Based on the results observed in Fig. 2(c)–(e), it is clear that the spectra observed from mixed-phase Pr^3+^:YPO_4_ also plays a critical role in the TAT-splitting. When *E*_1_ was fixed at 595 nm, the particle transfer occurs between ^1^D_2_ (γ_2_) → ^3^H_4_ (δ_0_) and level |1〉 splits into |+〉 and |−〉, and primary TAT-splitting is observed in Fig. 2(c1). The primary TAT-splitting can be explained from the dressing effect of the term |*G*_1_|^2^/*d*_1_ in eqn (2). In the ∧-type system, level |1〉 that is already split into |+〉 and |−〉 by *E*_1_ and when *E*_2_ was turned on, |−〉 will further split into |−+〉 and |−−〉 with the dressing effect of |*G*_2_|^2^/*d*_2_. Due to further splitting of |−〉, the secondary TAT-splitting would appear in Fig. 2(c2). By increasing *E*_2_ to 596.7 nm (particle transition between ^1^D_2_ (γ_1_) → ^3^H_4_ (δ_0_)), primary splitting becomes strong and the intensity of secondary peak decreases, while the splitting distance *Δ*_−±_ increases (Fig. 2(d2)), followed by a decrease in the dressing effect |*G*_2_|^2^/*Γ*_22_. When the wavelength of *E*_2_ is set to 600 nm, the secondary peak vanishes, and only primary TAT-splitting retains (Fig. 2(e2)). In the case of a two-level system, the primary TAT-splitting becomes very weak when observed at the third resonance point (Fig. 2(e1)). The three peaks of the hybrid time-domain signal (Fig. 2(c2)), from left to right, can be corresponded to the primary dressed state |−〉, the secondary dressed states |−+〉 and |−−〉, respectively, as shown in Fig. 1(e).

**Fig. 3:**
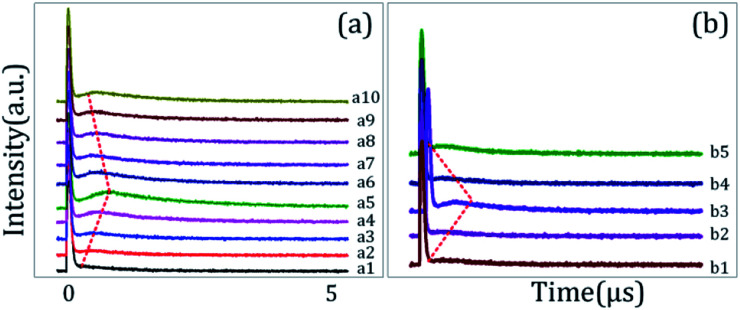
(a) and (b) show the evolution of the temporal intensity signal obtained from Pr^3+^:YPO_4_ in the two-level system when the detuning of *E*_1_ is changed, while *E*_2_ is blocked.

Fig. 3 shows the temporal intensity of the hybrid signal (FL + *E*_S_) in a two-level system by changing the detuning of *E*_1_ with blocked *E*_2._ When *E*_1_ is fixed at far off-resonant (*Δ*_1_ > 0), the temporal intensity signal has a very weak amplitude and has no AT splitting (Fig. 3(a1)). As *Δ*_1_ gets closer to the resonant wavelength, the amplitude of the intensity signal raises gradually due to an increase in the population transfer between ^1^D_2_ (γ_2_) → ^3^H_4_ (δ_0_) due to the resonant excitation of *E*_1_. At the resonance wavelength (*Δ*_1_ ≡ 0), a strong primary TAT-splitting is observed modelled by |*G*_1_|^2^/*d*_1_eqn (2), as shown in Fig. 3(a5). As the wavelength of *E*_1_ is further increased to the off-resonant wavelength, the primary TAT-splitting reduces (Fig. 3(a7)). The weak AT-splitting can be explained by the weak dressing effect of |*G*_1_|^2^/*d*_1_ due to the off-resonant excitation of the *E*_2_ beam. When the wavelength of *E*_1_ is further increased to 600 nm, the primary TAT-splitting increases (Fig. 3(b3)), which can be explained from an increase in the population transfer from ^1^D_2_ (γ_2_) → ^3^H_4_ (δ_2_). As detuning is further increased to off-resonant, the TAT-splitting disappears due to a very weak dressing effect, as shown in Fig. 3(b5).

The time-domain intensity of the hybrid signal in Fig. 3(a) shows the behaviour of the PNP transistor operating as a logic NAND gate. The model of the optical logic equivalent NAND gate is shown in Fig. 1(e). From Fig. 3, the right peak corresponds to the output logic 1, whereas the absence of the right peak corresponds to the output logic 0. In our proposed model of the logic NAND gate, “*A*” and “*B*” are two inputs on the time-intensity signal corresponding to either logic 0 or logic 1. When the wavelength of the input beam is changed to resonance, the logic input (*A*, *B*) satisfies the logical condition (1, 1) of the NAND gate, so the output of MOSFET is observed as OFF state (logical output 0) in Fig. 3(a1). When the wavelength is at off-resonance, the input *A* and *B* signal satisfies the logical condition (0, 0) of the NAND gate, and MOSFET output is observed to be in the ON state (logical output 1) in Fig. 3(a5). The output of the proposed NAND gate can be controlled through primary TAT-splitting. Our experiment results defined ON-state and OFF-state by the switching contrast *C* = (*I*_off_ − *I*_on_)/(*I*_off_ + *I*_on_), where *I*_off_ is the light intensity at the OFF-state and *I*_on_ is the light intensity at the ON-state.^18^ The switching contrast *C* is measured to be about 88% (Fig. 3(a5)). The total switching speed (20 ns) is taken to be the quadrature sum of several independent contributions.

**Fig. 4:**
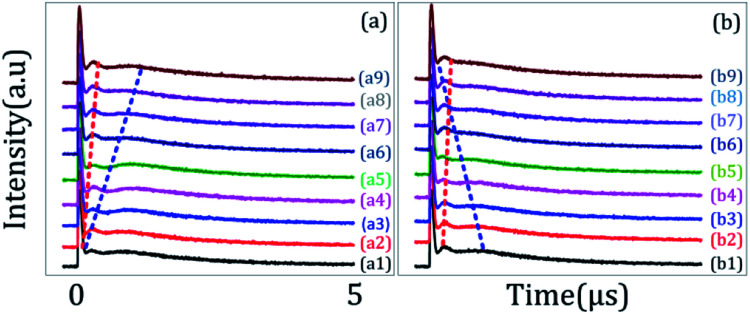
(a) and (b) shows the evolution of the temporal intensity signal obtained from Pr^3+^:YPO_4_ in the ∧-type three-level system when the detuning of *E*_1_ is fixed at resonant wavelength (*Δ*_1_ = 0), while *E*_2_ is changed.

Fig. 4 shows the excitation spectrum of the hybrid signal (FL + *E*_S_) in a ∧-type three-level system. The temporal intensity signal in Fig. 4 is measured by fixing *E*_1_ at resonance (*Δ*_1_ ≡ 0) and changing the detuning of *E*_2_. To observe the maximum TAT-splitting, the powers of both *E*_1_ and *E*_2_ were fixed at a high value (5 mW). Even when *E*_2_ is fixed at far off-resonance, very weak AT splitting is observed (Fig. 4(a1)). Such a weak AT-splitting can be explained from the dressing effect |*G*_2_|^2^/*d*_2_ caused by the high power of the *E*_2_ dressing beam. As *E*_2_ moved towards the peak position of the first spectral peak (595 nm), double TAT-splitting (both primary and secondary) is observed due to the dipole-allowed transitions between ^1^D_2_ (γ_2_) → ^3^H_4_ (δ_0_), as shown in Fig. 4(a4). The primary TAT-splitting results from the splitting of |1〉 into |+〉 and |−〉, whereas the secondary TAT-splitting comes from the further splitting of the primary dressed level |−〉 into two secondary dressed levels |−+〉 and|−−〉. The primary splitting distance (*Δ*_±_ = *λ*_+_ − *λ*_−_ = (*Δ*_12_ + 4|*G*_1_|^2^)^1/2^) between |+〉 and |−〉 and secondary splitting distance (*Δ*_−±_ = *λ*_−+_ − *λ*_−−_ = [*Δ*_2_ ± (*Δ*_22_ + 4|*G*_2_|^2^)^1/2^]/2) between the dressed levels |−+〉 and |−−〉 increases. Due to this, very clear double TAT-splitting is observed in Fig. 4(a4). When *E*_2_ moves towards the peak position of the second spectral peak (^1^D_2_ (γ_1_) → ^3^H_4_ (δ_0_)), secondary splitting distance (*Δ*_−±_) reduces and becomes very weak (Fig. 4(a9)). At this wavelength, the intensity of the primary TAT-splitting is observed to be maximum. When the wavelength of *E*_2_ is further increased to 598 nm, the TAT-splitting becomes very weak (Fig. 4(b5)), followed by off-resonant excitation at this stage. It is interesting that even at the far resonant wavelength single AT-splitting is observed (Fig. 4(b9)).

The multi-channel optical routing was realized by the primary and secondary TAT-splitting results observed in Fig. 4. Our experiment provides a physical mechanism to realize all optical routing in real-time by controlling laser detuning. Furthermore, we can see the division of one peak (Fig. 4(a1)) into two peaks (Fig. 3(a5)) due to change in laser detuning from off-resonant to resonant. Therefore, the corresponding switching ratio of our routing model is about 2. In our experiment, the channel equalization ratio can be defined as^28^
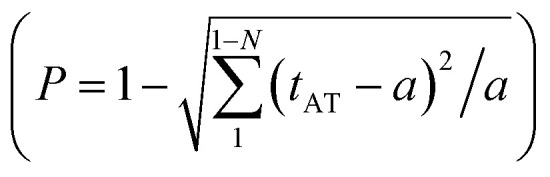
 where “*a*” and “*a*_*i*_” is the area of one peak and gap between the neighbouring peaks, respectively. In our experiment, the channel equalization ratio *P* increases up to 91% (Fig. 4(a4)) as the detuning of the input beam is changed from 590 nm to 596.7 nm. For temporal signals, contrast index can be defined as *η* = (*t*_AT_ − *t*)/(*t*_AT_ + *t*), where (*t*_AT_*− t*) is the splitting between two neighbour peaks then, higher the contrast index, greater accuracy of information and less crosstalk between channels. From our experiment, we measured *η* = 87% (greater accuracy of the information and less crosstalk between channels), and the average power of our routing can be operated at 1–2.0 μW.

**Fig. 5:**
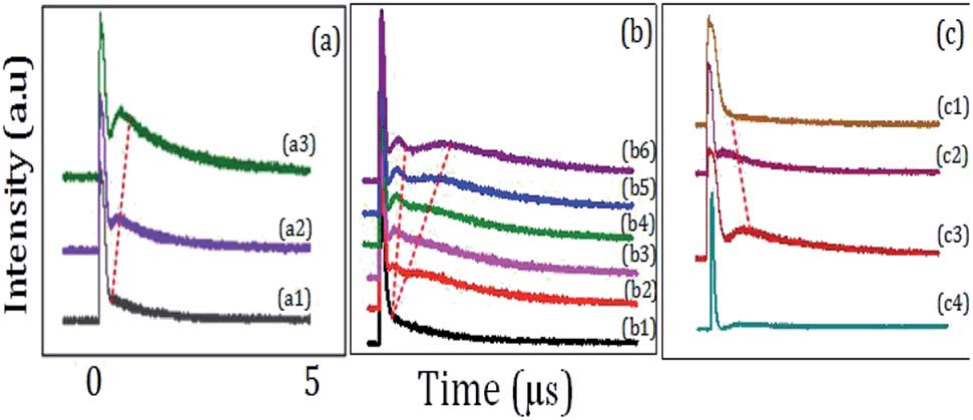
(a) shows the evolution of the TAT-splitting with the double dressing condition when power of *E*_1_ is increased from 1 mW to 4 mW (*E*_2_ is blocked). (b) shows the evolution of primary and secondary TAT-splitting from (H + T)-phase Pr^3+^:YPO_4_, when power of *E*_2_ is increased from 1 mW (bottom) to 8 mW (top) and *E*_1_ is fixed at 3 mW (c) shows evolution of TAT-splitting with double dressing condition when *E*_1_ and *E*_2_ are fixed at resonant wavelengths for (c1) H-phase Pr^3+^:YPO_4_, (c2) T-phase Pr^3+^:YPO_4_, and (c3) (T + H)-phase Pr^3+^:YPO_4_, (c4) (H + T)-phase Eu^3+^:YPO_4_.

Fig. 5(a1)–(a3) show TAT-splitting in a hybrid signal due to the dressing effect of *E*_1_ with the increase in the splitting distance from bottom to top peaks as the power of *E*_1_ is increased from low to high in (H + T)-phase Pr^3+^:YPO_4_. At first, the power of *E*_1_ is too little (1 mW) to split |1〉 into dressed energy levels, so we cannot see the right peak appearing in Fig. 5(a1). With an increase in the power of *E*_1_ (≈4 mW), the energy level |1〉 splits into |±〉, and primary AT-splitting appears in Fig. 5(a3). We can explain that the primary TAT-splitting distance (*Δ*_±_ = *λ*_+_ − *λ*_−_ = (*Δ*_1_^2^ + 4|*G*_1_|^2^)^1/2^) between |+〉 and |−〉 is directly proportional to the power of *E*_1_. With an increase in *E*_1_, |*G*_1_|^2^ keeps increasing, which leads to an increase in the splitting distance, and prominent primary TAT-splitting was observed in Fig. 5(a3).

Fig. 5(b) shows the time-domain intensity of the hybrid signal by fixing *E*_1_ and *E*_2_ at resonant wavelengths and changing *P*_2_ of *E*_2_ in a ∧-type three-level system. In a ∧-type system, when power was changed from 1 mW (Fig. 5(b1)) to 4 mW (Fig. 5(b3)), the primary TAT-splitting follows a similar trend as explained for the two-level system (Fig. 5(a)). With an increase in the power of *E*_2_ (≈8 mW), the primary dressed level |−〉 further split into two secondary dressed levels |−+〉 and |−−〉, as shown in Fig. 1(d), and the secondary AT-splitting is observed in Fig. 5(b5). The secondary distance (*Δ*_−±_ = *λ*_−+_ − *λ*_−−_ = [*Δ*_2_ ± (*Δ*_2_^2^ + 4|*G*_2_|^2^)^1/2^]/2) between the primary and secondary peak in the time-domain is only determined by *E*_2_. By increasing the power of *E*_2_, |*G*_2_|^2^ increases, which leads to an increase in *Δ*_−±_. The observation of the primary and secondary dressing results from the exhibition of the strong atomic-like behavior of the mixed-phase Pr^3+^:YPO_4_ due to a strong transfer probability of T-phase and low symmetry of H-phase. Hence, the prominent secondary peak is observed at high power (Fig. 5(b6)).

Fig. 5(c1), (c2), and (c3) shows the time-domain signal obtained from the H-phase Pr^3+^:YPO_4_, mixed (T + H)-phase Pr^3+^:YPO_4_, and T-phase Pr^3+^:YPO_4_, respectively. The time-domain signal is obtained under the same experiment condition as defined for Fig. 5(a). From Fig. 5(c1) and (c2), it can be clearly seen that no TAT-splitting is observed for H-phase Pr^3+^:YPO_4_ (Fig. 5(c1)), whereas (T + H)-phase Pr^3+^:YPO_4_ (Fig. 5(c3)) has strongest TAT-splitting among the three samples. Hexagonal-phase Pr^3+^:YPO_4_ has a low *D*_2_ point-group symmetry site, which results in the strong atomic-like behavior. The hexagonal-phase Pr^3+^:YPO_4_ should have a strong dressing effect, but no TAT-splitting is observed (Fig. 5(c1)). This can be explained from the low transfer probability and weak dipole moment, which results in a weak dressing effect in the H-phase Pr^3+^:YPO_4_, as shown in Fig. 5(c1). Unlike H-phase Pr^3+^:YPO_4_, the T-phase Pr^3+^:YPO_4_ has the high *D*_2d_ point-group symmetry, which results in a relatively weak atomic-like behavior and should have a weak dressing effect. Due to strong transfer probability and dipole moment in the T-phase Pr^3+^:YPO_4_, comparatively strong TAT-splitting is observed in Fig. 5(c2). Meanwhile, the mixed-phase (T + H) Pr^3+^:YPO_4_ demonstrates the strongest TAT-splitting among the three samples, as shown in Fig. 5(c1). YPO crystal having mixed (T + H)-phase occupy (*D*_2_ + *D*_2d_) site symmetry, which combines the strong transfer probability and dipole moment of the T-phase with strong atomic-like behavior of the H-phase Pr^3+^:YPO_4_. Hence, strong TAT-splitting was observed in (much-T + less-H)-phase, as shown in Fig. 5(c3). In the mixed-phase (much-H + less-T) Pr^3+^:YPO_4_ (Fig. 5(b)), both primary and secondary dressing is observed as compared to the observation of only primary dressing in (H + T)-phase Pr^3+^:YPO_4_ (Fig. 5(c3)). This can be explained from the stronger atomic-like behavior of the H-phase as compared to Fig. 5(c3).

To study the effect of different doped ion on TAT-splitting, next, we compared (H + T)-phase Pr^3+^:YPO_4_ (Fig. 5(b)) with (H + T)-phase Eu^3+^:YPO_4_ (Fig. 5(c4)). Fig. 5(c4) shows the time intensity of the hybrid signal obtained from (H + T)-phase Eu^3+^:YPO_4_. By comparing Fig. 5(b6) with Fig. 5(c4), we can conclude that TAT-splitting is stronger in Pr^3+^ ion than in Eu^3+^. This can be explained from the higher dipole moment of Pr^3+^:YPO_4_ as compared to Eu^3+^:YPO_4_, which corresponds to a stronger dressing effect.

Here, the optical MOSFET equivalent NAND gate has been realized in Fig. 5(a) and (c)). The model of MOSFET logic equivalent NAND gate is shown in Fig. 1(f), where *E*_1_ and *E*_2_ are the input signal, and *Y* is the output of the MOSFET. To realize the switching function of the MOSFET, when the power of *E*_1_ is changed, the temporal intensity satisfies the logical condition (1, 1) of the NAND gate, and the output of the MOSFET *Y* performs OFF-state as a spectral peak in Fig. 5(a1, c1, and c4). Here, the output of the MOSFET *Y* satisfies the logical 0 condition. The temporal intensity input spectral signal satisfies the logical condition (0, 0) of the NAND gate, and the output of the MOSFET *Y* performs ON-state as a spectral peak in Fig. 5(a2), (a3), (c2), and (c3). Here, the output of the MOSFET *Y* satisfies the logical 1 condition, where the switching contrast *C* is about 85% from Fig. 5(a1) to Fig. 5(a3). Based on the primary and secondary TAT-splitting, one can exploit this as a multi-channel optical router. In the first stage of the three-level atomic system, one channel is converted into intermediate state two-channel, where the channel equalization ratio (*P*) increases from 15% (Fig. 5(b1)) to 50% (Fig. 5(b2)), and the contrast index rises to *η* = 55% (Fig. 5(b2)) as laser power is increased from 1 mW to 3 mW. In the second stage, when the power of *E*_1_ is increased to 8 mW, the intermediate state two channels are successfully converted in to complete two-channel, and the channel equalization ratio (*P*) increases to 93% (Fig. 5(b6)) with the contrast index rising up to *η* = 95% (Fig. 5(b6)). In comparison to laser detuning (Fig. 4), the higher channel equalization ratio and contrast index is measured with respect to power. This can be explained from an increase in the dressing effect at high power. Hence, the routing channels became more distinguishable, as shown in Fig. 5(b6).

## Conclusion

In summary, we have demonstrated the primary and secondary TAT-splitting from different phases of the Pr^3+^:YPO_4_ crystal. We observed that the site symmetry-dependent TAT-splitting from mixed phases (H + T) had a strong dressing effect than that from the pure T-phase or H-phase. Also, we discussed the dressing effect dependency on the site symmetry of different ions (Eu^3+^ and Pr^3+^) in the host material YPO_4_. We also observed that the Pr^3+^ ion had stronger TAT-splitting than Eu^3+^ ion in the host material of YPO. Further, the TAT-splitting depends on the distance between bright states caused by the dressing effect. The splitting distance increases, if laser power increases, and the time delay becomes larger. The primary and secondary AT-splitting were caused by single and double dressing, respectively, and mainly depends upon the electric dipole transition and power of laser beams. The multi-channel optical router and logic NAND gate were also realized from the TAT-splitting. The channel equalization ratio, contrast index, and switching contrast were controlled by laser detuning and the power of input beams.

## Conflicts of interest

There are no conflicts to declare.

## Supplementary Material
